# The Association of Foot Arch Variations With Patellofemoral Pain Syndrome in Recreational Athletes

**DOI:** 10.1177/23259671251334776

**Published:** 2025-06-03

**Authors:** Ankit Gudi, Ashish John Prabhakar, Charu Eapen, Vijayakumar Palaniswamy, Yogeesh D. Kamat

**Affiliations:** * Department of Physiotherapy, Kasturba Medical College Mangalore, Manipal Academy of Higher Education, Karnataka, Manipal, India; ‡ Department of Orthopedics, Kasturba Medical College Mangalore, Manipal Academy of Higher Education, Karnataka, Manipal, India; Investigation performed at Kasturba Medical College Mangalore, India

**Keywords:** patellofemoral pain syndrome, anterior knee pain, athletic injuries, flat foot

## Abstract

**Background:**

Patellofemoral pain syndrome (PFPS) is one of the common causes of anterior knee pain, especially in the young and athletic population. Although there are many intrinsic and extrinsic factors causing PFPS, the foot arch variations have been theorized to be associated with or contribute to the condition.

**Purpose:**

To study the association of foot arch variations with PFPS in recreational athletes.

**Study Design:**

Cross-sectional study; Level of evidence, 3.

**Methods:**

This study investigated the foot arch index, categorizing the foot as flat foot, high-arch foot, or normal foot in 35 recreational athletes with PFPS and 35 healthy individuals (12 women, 23 men in each group). The foot posture index was calculated by taking the impression of the foot on a paper using ink and uploading it on a computer to determine the area of the foot. The midfoot area was then divided by the total area to obtain the foot arch index. Based on this, the foot was categorized as flat foot, high-arch foot, or normal foot.

**Results:**

Using the chi-square test, the authors observed that there were significantly more individuals with foot arch variations in the PFPS group as compared with the healthy individuals. A chi-square test was used to study the association between the foot arch variations and PFPS and found a significant (*P* < .001) association between the two, and a post hoc comparison showed a significant (*P* < .001) association of flat foot with PFPS.

**Conclusion:**

A more pronated or flat foot is associated with PFPS and should be assessed when evaluating individuals with PFPS. Hence, the evaluation of foot arch may be an important addition to other clinical measurements taken to explore the factors associated with PFPS.

**Registration:**

CTRI/2024/01/061538 (Clinical Trials Registry–India code).

Patellofemoral pain syndrome (PFPS) is a prevalent source of knee pain among adolescents and young adults.^1^ It is defined as pain occurring in the prepatellar or retropatellar region that worsens during prolonged sitting, kneeling, squatting, and stair climbing. PFPS is a frequent cause of anterior knee pain and mainly affects young athletes without any structural abnormalities.^14^ The term “anterior knee pain” has been proposed to cover all pain-related issues, excluding conditions such as intra-articular pathology, peripatellar tendinitis or bursitis, plica syndromes, Sinding-Larsen disease, Osgood-Schlatter disease, neuromas, and other infrequent pathologies. By excluding these conditions, it has been suggested that patients who still have anterior knee pain could be clinically diagnosed with PFPS.^21^

The incidence of anterior knee pain is high, affecting 22 in 1000 persons per year.^14^ Increased engagement in competitive and recreational sports in recent years has raised awareness of overuse knee problems.^23^ Athletes playing sports that involve running and jumping, such as basketball or tennis, have been reported to be the most affected population. According to a recent study, approximately 25% of recreational athletes with PFPS will stop playing sports due to knee pain.^14^

A recreational athlete is an individual who participates in sports activities involving stop-jump tasks like basketball, soccer, and volleyball on an infrequent basis—typically ≤3 times a week—and does not adhere to a structured training program designed by a professional.^4^ He or she participates in sports to stay physically healthy, be active in society, and, most of all, have fun.

Identified potential risk factors for PFPS encompass various factors such as weakness in the gastrocnemius, hamstring, or quadriceps; tightness in the iliotibial band; general ligament laxity; insufficient hamstring or quadriceps strength; weakness in the hip musculature; compression of the patella; and abnormal reflex timing between the vastus lateralis and vastus medialis muscles.^24^ Patellar characteristics, muscle activation time, and Q-angle are also some of the other major risk factors.^9^ External factors include overtraining and inappropriate shoes.^2^ The pathomechanics of pain in patellofemoral syndrome is theorized to be the delay in the activity of the vastus medialis oblique and vastus lateralis muscles relative to each other, which causes abnormal displacement of the patella to the side. This abnormal displacement of the patella, known as patellar malignment, causes increased pressure over the patellofemoral joint (PFJ), leading to pain.^8^

In clinical settings, static foot posture is frequently assessed because of the suggested connection between foot alignment and lower limb injuries, with the aim to identify potential indications for biomechanical interventions.^16^ Foot pronation and lower extremity conditions, such as medial tibial stress syndrome, patellofemoral pain, and exercise-associated lower extremity injuries, have also long been theorized to be connected.^12^ A more pronated foot type causes excessive or prolonged pronation of the foot during gait, resulting in greater tibial and femoral internal rotation.^15^ Because of the increased internal rotation of the femur under the patella, these kinematic abnormalities may result in increased lateral PFJ stress.^18^ A recent systematic analysis examining gait-related kinematics revealed that individuals experiencing PFPS might exhibit delayed-peak rearfoot eversion and heightened rearfoot eversion at the moment of heel strike.^22^ Research conducted by Tong and Kong^22^ concluded that both pronated and supinated foot types are notably linked with lower limb injuries. However, the reliability of the study was deemed low, and the authors were unable to provide detailed insights into specific pathologies and outcome measures.

Flat feet have been linked to the development of anterior knee pain and occasional episodes of low back pain.^7^ Moderate to severe flat feet are associated with nearly double the incidence of anterior knee pain and intermittent low back pain.^7^ Because this study had a retrospective design and was based on co-relational data, it was very weak.

In recent years, some studies have used foot orthoses as a single treatment for PFPS, and others have chosen foot orthoses combined with physical therapy or exercise therapy.^5^ Regarding the connection between PFPS and foot and ankle variations, conflicting data have been found. Furthermore, the lack of prospective studies and the moderate quality of protocols call for larger studies with a higher level of evidence to determine whether foot position and arches are associated with PFPS.

## Methods

### Study Design

This was a cross-sectional study conducted at KMC Mangalore hospitals (Ambedkar Circle and Attavar) between January 2023 and May 2024. This study was time-bound, and it received ethics approval from the KMC Mangalore, Manipal Academy of Higher Education’s Institutional Ethics Committee (IEC KMC MLR-01/2023/22). It was registered in India’s clinical trial registry (code CTRI/2024/01/061538). Informed consent was obtained after explaining the details of the study to all the participants.

### Participants

In this study, 70 participants were recruited, of whom 35 had PFPS and 35 did not. The sample size was calculated using a reference from a previous study using similar variables for the *r* value calculation. Inclusion criteria for the experimental group included recreational athletes between 18 and 30 years of age diagnosed with PFPS by an orthopaedic doctor and with pain provoked by at least 2 activities: running, walking, hopping, squatting, stair climbing, kneeling, or prolonged sitting. The control group consisted of age- and sex-matched participants without PFPS. Exclusion criteria included a history of previous surgeries of lower extremity musculoskeletal structures, including bone, ligaments, and/or nerve injury; any acute injury to musculoskeletal structures of the lower limb, either sprain, strain, or fracture in the last 3 months; concomitant injury or pain arising from the lumbar spine or hip; knee internal derangement; and early osteoarthritis.

### Procedure

**Figure 1.**
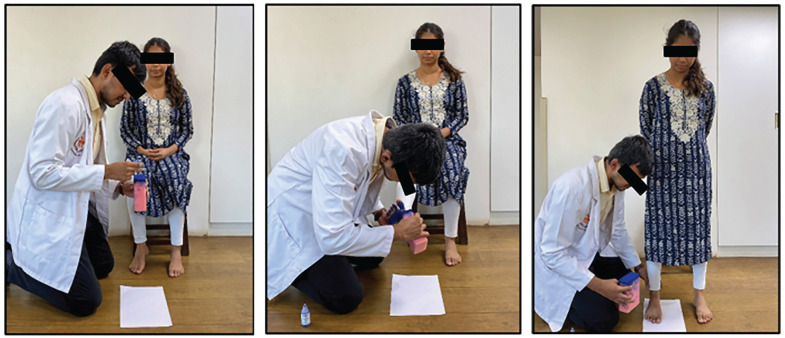
Coloring the foot with ink and transferring weight to the inked foot kept on paper.

**Figure 2.**
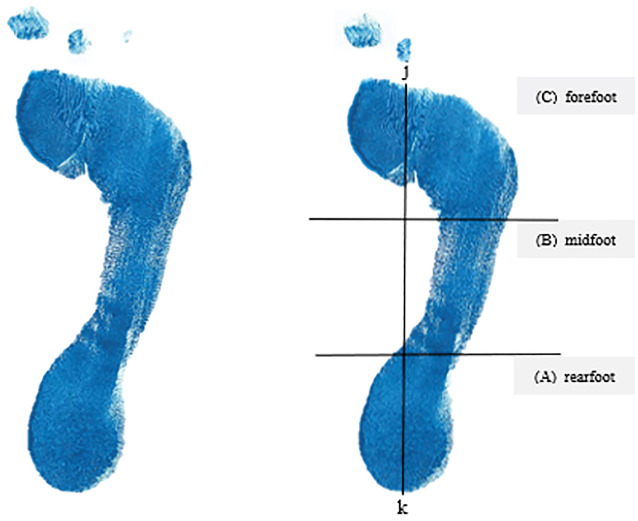
Footprint analysis.

Considering the inclusion and exclusion criteria, individuals with PFPS and healthy individuals were recruited. The goal of the study was communicated, and those who were willing to participate were asked to sign an informed written consent form. Demographic data and assessments were collected from the included participants. The standard protocol for measuring the foot arch index described by Cavanagh and Rodgers^3^ was used, which states that the arch index lying within the range of 0.21 to 0.26 is to be categorized as a normal-arch foot, whereas the arch index <0.21 is categorized as a high-arch foot and >0.26 is categorized as a flat-arch foot. The footprint was taken during the half body weight stance. While the participant had one foot on the ground, the other foot was inked (Figure 1, A and B). Some weight was gradually transferred to the inked foot on the paper until one-half of the body weight was transferred (Figure 1C). The footprint (Figure 2A) was then digitalized by scanning and uploading on a laptop. The digital plantar footprint was analyzed with AutoCAD software (Autodesk) to calculate the arch index, and markings were made to make measurements.

Marking of the footprint started with a line drawn from the center of the heel (point *k* in Figure 2B) to the tip of the second toe. This line was called the foot axis. A second line perpendicular to the axis was then drawn such that it was tangential to the most anterior part of the outline of the main body of the footprint in front of the metatarsal heads. The point of intersection between these 2 lines was marked (point *j* in Figure 2B). The line *jk* was then divided into equal thirds, as shown in Figure 2B. A perpendicular line to the foot axis was drawn at each 33.3% mark along *jk*, dividing the foot into the rearfoot (*A*), midfoot (*B*), and forefoot (*C*) regions. The total area of the footprint (*A* + *B* + *C* in Figure 2B) and the area in the midfoot (*B* in Figure 2B) were then determined. The arch index was then calculated by dividing *B* by (*A* + *B* + *C*). Based on this calculation, the foot was then categorized as either flat foot, high-arch foot, or normal foot.

### Statistical Analysis

The collected data were entered into Statistical Package for Social Sciences (SPSS) Version 25 (IBM Corp). The association between PFPS and foot arch variations was tested using the chi-square test at the .01 level of significance. Chi-square testing was used because it tests the goodness of fit of the 2 groups of data—participants with PFPS and participants without PFPS—which were a set of nominal data. Additionally, to identify which type of foot is more associated with PFPS, post hoc comparison using Bonferroni was done, as this provides a precise result of the association.

## Results

**Figure 3.**
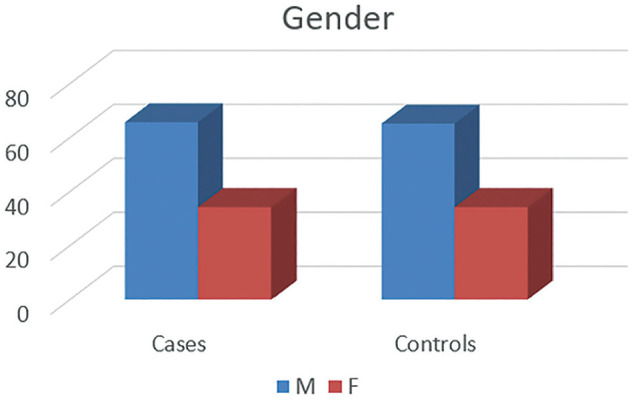
Number of male and female participants in each group.

In the present study, 70 recreational athletes were recruited (mean age, 23.09 ± 2.32 years), of whom 35 had PFPS and 35 were healthy individuals. Of the 35 participants in each group, 23 were men and 12 were women (Figure 3). All the participants of the study were involved in stop-jump and running sports like badminton and football and played recreationally.

### Foot Area

**Figure 4.**
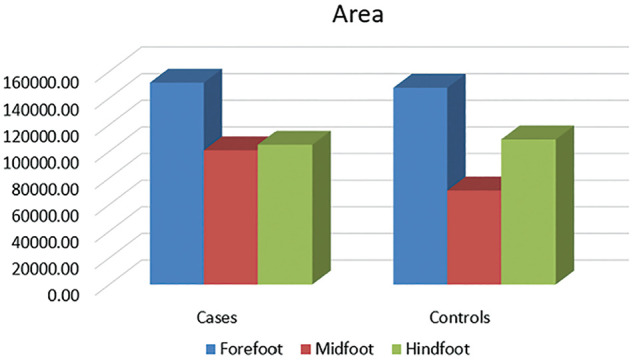
The foot area (in pixels) measured in 3 divisions: (1) forefoot, (2) midfoot, and (3) hindfoot.

The foot area (in pixels) was measured in 3 divisions: (1) forefoot, (2) midfoot, and (3) hindfoot (Figure 4).

The mean forefoot area (in pixels) for the PFPS group was 156,322.6 ± 46,968.45, whereas for the control group it was 149,471.17 ± 30,931.47. The mean midfoot area (in pixels) for the PFPS group was 86,274.91 ± 49,204.05, whereas for the control group it was 73,301.94 ± 21,931.18. The mean rearfoot area (in pixels) for the PFPS group was 96,562.49 ± 35,504.21, whereas for the control group it was 100,123.86 ± 24,134.38.

### Foot Arch Index

**Figure 5.**
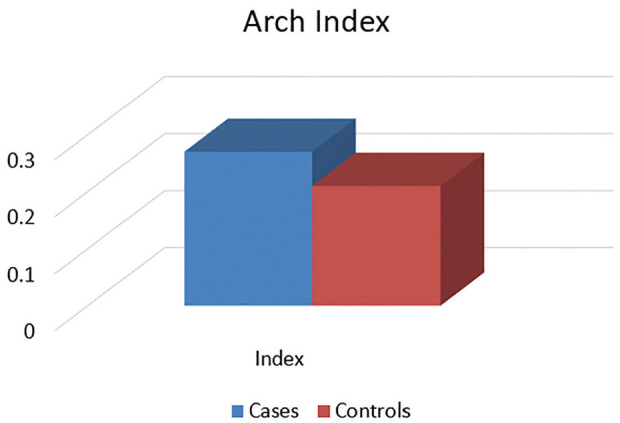
Arch index.

The foot arch index calculated by the footprint method was 0.27 ± 0.08 for the PFPS group and 0.21 ± 0.04 for the control group (Figure 5).

### Type of Foot

**Figure 6.**
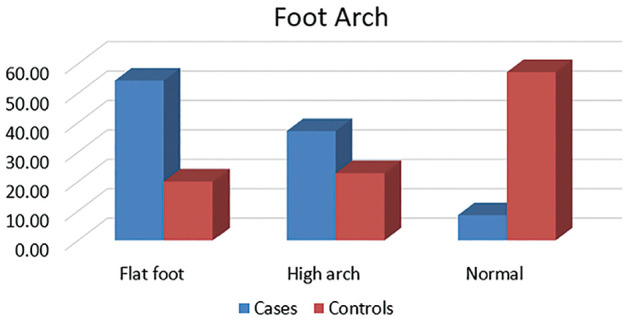
Foot arch.

Of the 35 participants diagnosed with PFPS, 19 (54.29%) participants were found to have a flat foot, 13 (37.14%) had a high-arch foot, and 3 (8.57%) had a normal-arch foot (Figure 6). In the control group, 7 (20%) had a flat foot, 8 (22.86%) had a high-arch foot, and 20 (57.14%) had a normal-arch foot.

The distribution of individuals in the PFPS and control groups in the 3 foot categories is shown in Table 1. The chi-square test showed a positive association between the foot arch variations and PFPS in the investigated population (Table 2).

**Table 1:** Contingency Table*^a^*

PFPS	Type of Foot			Total
	Flat Foot	High Arch	Normal	
No	7	8	20	35
Yes	19	13	3	35
Total	26	21	23	70

a PFPS.

**Table 2:** Chi-Square Test Results

	Value	df	*P*
χ^2^	19.3	2	<.001
N	70		

Additionally, to identify which type of foot is more associated with PFPS, post hoc comparison using Bonferroni showed a significant (*P* = .001) association of PFPS with a high-arch foot and a highly significant (*P* < .001) association of PFPS with a flat foot (Table 3).

**Table 3:** Post Hoc Comparison: Type of Foot

Type of Foot	Type of Foot	Difference	SE	*t*	df	*P*_Bonferroni_
Flat foot	High arch	0.112	0.128	0.875	67.0	>.999
Flat foot	Normal	0.600	0.125	4.822	67.0	<.001
High arch	Normal	0.489	0.131	3.722	67.0	.001

## Discussion

Patellofemoral pain is a commonly encountered condition of the lower limb, often affecting young, physically active individuals. PFPS has been associated with poor control of the distal segments of the limb, such as the foot structure, which is believed to alter loading stress within the PFJ.

The objective of this study was to examine the association of foot arch variations with PFPS using the footprint method. A total of 70 recreational athletes were recruited for the study, of whom 35 were diagnosed with PFPS and the other 35 were healthy individuals. Of the 35 participants with PFPS, 23 were men and 12 were women, which is not in line with the findings of previous literature stating that females have a 2.23 times higher incidence of PFPS compared with males. This sex incongruity in PFPS incidence is attributed to various biomechanical and anatomic alignment factors. Females, in comparison with males, typically exhibit increased static measurements of the Q-angle; heightened dynamic measures of the knee valgus angle, hip internal rotation angle, hip adduction moment, and knee valgus moment; decreased dynamic measurements of the knee flexion angle; and reduced strength in the quadriceps, hip external rotation, hip extension, and hip abduction strength. These deficiencies in females are theorized to serve as risk factors for PFPS, leading many researchers to propose that females have a higher prevalence and incidence of PFPS. But the increased number of cases of PFPS in men in the present study might be because of the higher participation of men in recreational sports in our study setting. It was observed that a greater number of male recreational athletes participated in sports that involved running and/or jumping, like basketball, badminton, volleyball, and football, which are subject to causing PFPS in these athletes. The difference in our results as opposed to the results from the existing literature may also be due to the possibility that, with respect to female recreational athletes, significantly more male recreational athletes have reported musculoskeletal injuries than unreported musculoskeletal injuries. It can be hypothesized that this is also a result of the difference in the sexes participating in these sports. Additionally, women with PFPS who are not involved in any kind of sport are most likely not going to participate in sports due to the high activity demands that will be placed on them.

Various methods have been used to determine the foot type, like the foot arch index using the footprint, navicular drop, foot posture index, resting calcaneal measure, and longitudinal arch angle. Footprint parameters have been suggested to be valuable and reliable indicators of arch height in gait-related studies. Among these parameters, the arch index has garnered significant scientific interest and high validity, enabling researchers and practitioners to categorize static arch formations as high, low, or normal. In the current study, the foot arch index was noted to be within the normal range for the control group, whereas for the PFPS group, it was seen in the higher range, which is suggestive of the association between PFPS and foot arch variations.

Foot pronation, resulting in inward rotation of the tibia and femur, can increase the reaction forces within the PFJ, leading to higher loads on the joint. Because activities like running and jumping typically impose significant loads on the PFJ, athletes with PFPS who display a more pronated foot position may be prone to lower extremity injuries during sports-related activities.^22^

The calcaneal eversion would move the ankle joint mortise such that a medial deviation of the tibia occurs and produces an increase in the knee valgus. This observation aligns with previous findings indicating that increased eversion of the foot correlates with heightened knee valgus during weightbearing.^6^ Changes in limb alignment, particularly an increase in knee valgus, can influence the distribution of load on the PFJ, generating uneven stresses on the facets of the patella and the underlying femoral trochlear groove. This not only leads to patellofemoral pain but also potentially predisposes the PFJ to osteoarthritis.

Excessive pronation during midstance leads to an exaggerated internal rotation of the tibia.^13^ To facilitate knee extension, the tibia needs to externally rotate in relation to the femur to ensure proper movement for the screw-home mechanism. However, when abnormal or excessive pronation inhibits sufficient tibial external rotation, the femur compensates by internally rotating on the tibia, facilitating the required rotation for extension. This internal rotation of the lower limb caused by pronation of the subtalar joint results in an increase in the Q-angle and the lateral component of the quadriceps vector. An elevated Q-angle due to excessive foot pronation also predisposes the knee to PFPS.^20^

Foot pronation at the subtalar joint also plays a crucial role in managing the effect of ground-reaction forces while walking. When there is an excessive amount of pronation, it is suggested that the smaller intrinsic foot muscles and larger extrinsic muscles engage for longer periods, particularly during eccentric contractions. This prolonged engagement can lead to muscle fatigue setting in earlier, causing more force to be absorbed by the periosteum, which is a dense, fibrous connective tissue sheath that covers the bones, potentially increasing the risk of overuse injuries. Richie and colleagues^19^ investigated the differences in muscle activity between eccentric and concentric contractions in 10 runners across 3 surfaces with varying hardness. Their study revealed that running on hard, rigid surfaces like concrete prompted higher activity in the eccentric medial shin, which eventually led to fatigue. This emphasizes the importance of intrinsic shock absorption, accentuating eccentric muscle activity, and its potential contribution to the development of certain conditions. Consequently, it is probable that an increased amount of force is absorbed by the periosteum and bone, potentially contributing to the onset of PFPS. Certain high-risk groups, such as women or individuals with a pronated foot type, might benefit from using shoe inserts or orthoses to mitigate intrinsic shock absorption, thereby reducing the force exerted on the tenoperiosteum and bone.^26^

In the investigated population, we noted that there was a significant association between individuals with flat foot and PFPS. The existing literature states that there is controversy regarding the type of foot and its contribution to PFPS. Powers and colleagues^17^ in their study reported that a greater degree of rearfoot varus was observed in the individuals with PFPS as compared with the non-PFPS group. Likewise, subtalar inversion and rearfoot valgus were observed to be higher in a relaxed standing posture among study participants with PFPS in a study done by Levinger and Gilleard.^10^ In contrast, in their study, Messier et al^11^ concluded that individuals with PFPS had a normal-arch foot. Witvrouw et al^25^ also investigated the foot types in individuals with and without PFPS and found no significant differences.

### Limitations

This study is not without limitations. The sample was predominantly male recreational athletes with PFPS, whereas PFPS is more common in women; therefore, caution should be taken in generalizing the findings to the entire population of recreational athletes. The results of our study suggest that flat foot is associated with PFPS, but other factors that may be associated with PFPS were not controlled in this study, which is another limitation.

## Conclusion

The findings of the current investigation suggest that there exists a significant association between foot arch variations and PFPS, of which flat foot is significantly more associated with PFPS among the 3 types of foot.
